# History and Current State of Radiation Oncology Services and Practice in Jordan

**DOI:** 10.1200/GO.20.00074

**Published:** 2020-06-18

**Authors:** Jamal Khader, Abdelatif Al Mousa, Samir Al-Kayed, Hana Mahasneh, Rasmi Mubaidin, Nabeel Al Nassir, Sami Khatib, Adnan Qasem, Inad Haddadin, Elayan Elayan, Sondos Al Khatib

**Affiliations:** ^1^ Radiation Oncology Department, King Hussein Cancer Center, Amman, Jordan; ^2^ Al Afia Radiotherapy and Nuclear Medicine Center, Amman, Jordan; ^3^ Radiation Oncology Department, Queen Alia Hospital, Royal Medical Services, Amman, Jordan; ^4^ Radiotherapy Department, Al Basheer Hospital, Ministry of Health, Amman, Jordan; ^5^ Medical Physics Section, Radiation Oncology Department, King Hussein Cancer Center, Amman, Jordan

## Abstract

Radiation therapy (RT) for patients with cancer in Jordan began with a small individual effort and has now grown to be one of the most prominent treatment centers in the Middle East. Currently, there are 4 different centers that provide RT not only for the people of Jordan but also for citizens of other neighboring Arab countries. Because Jordan is a developing country, it still faces problems with the insufficient number of RT machines available and their supporting staff (physicists and technologists among others). In this article, we shed light on the history and current status of radiation oncology in Jordan and discuss the challenges we face.

## INTRODUCTION

Jordan is a Middle Eastern country with limited resources and economic constrains. Jordan is divided into 12 governorates and occupies an area of approximately 89,000 km^2^ with a 2018 estimated population of 10 million.^1^ Cancer is the second leading cause of death in Jordan after cardiovascular disease.^2^ Cancer care is delivered through a combination of government, non- government, academic, and private sectors without any national guidelines concerning treatment protocols or quality assurance. Most services are concentrated in the capital city of Amman, except for King Abdullah University Hospital in the north. Despite several initiatives, Jordan does not have a national cancer control plan. In countries in which cancer control programs have been implemented, the burden of cancer is decreasing and treatment outcomes are improving, which supports the need for Jordan to pursue similar strategic goals.^3^ In Jordan, the government bears the cost of cancer treatment. Cancer treatment is offered to Jordanians at no cost through government hospitals and is supported by agencies such as the Ministry of Health (MOH), Royal Medical Services (RMS; military), university hospitals, and the King Hussein Cancer Center (KHCC), which is a non-governmental facility. Recently, KHCC and the King Hussein Cancer Foundation (KHCF) took the lead in changing the insurance landscape for patients with cancer by introducing a nonprofit cancer insurance program that partially covers the cost of cancer care at KHCC for program participants who pay affordable premiums. The major private insurance companies do not cover cancer screening or treatment. A small minority of patients travel to Europe or the United States for treatment. According to data from the Jordan Cancer Registry in 2015, 8,400 patients were diagnosed with cancer: 5,556 Jordanians and 2,844 non-Jordanians.^4^

**CONTEXT**

**Key Objective**
To explain when and how radiation therapy (RT) began in Jordan and how it has evolved over the years.
**Knowledge Generated**
Since the late 1950s, efforts have resulted in an ever-growing RT practice in Jordan. As a result, 4 cancer facilities with cutting-edge technology have been successfully established.
**Relevance**
Jordan, although it is a country with limited resources, can overcome the obstacles and challenges it faces to deliver cancer care and RT efficiently for citizens of Jordan and those of neighboring countries.

**FIG 1:**
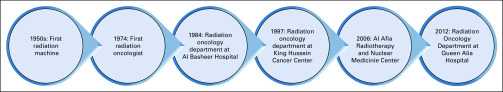
Milestones of radiation oncology in Jordan.

Non-Jordanian patients with cancer who are treated in Jordan pay their own expenses; they are covered either by their own governments or by charitable organizations that have agreements with the treating institutions. Lung, colorectal, bladder, and prostate cancer and lymphoma are the most common cancers in men, and breast, colorectal, thyroid, and uterine cancer and non-Hodgkin lymphoma are the most prevalent cancers among women.^4^ It is estimated that 50% to 60% of patients with cancer will need radiation therapy (RT) as part of their overall treatment. The practice of RT in Jordan has achieved many milestones (Fig 1).

### The First Treatment Machine in Jordan

Jordan’s first private hospital was established by a physician named Qasem Malhas in 1945.^5^ A few years later, that hospital acquired Jordan’s first radiation machine (10-100 kV), which was used for treating superficial tumors. At that time, radiologists were treating patients with help from radiation physicists and technologists. No single radiation oncologist was available in Jordan.

In 1962, Doctor Malhas gave the Jordanian MOH a radiation machine as a gift. The machine was then transferred to Al Basheer Hospital, which is the main governmental hospital in Amman. The hospital used the machine for managing superficial tumors until the MOH managed to acquire an Ortho-voltage machine (300 kV, Maxi mar x-ray) in 1964. At this point, nuclear medicine specialists along with radiation physicists and technologists began to take part in patient management. A few years later in 1967, the radiology department at the hospital was expanded once more with the arrival of its first external beam radiotherapy (EBRT; teletherapy) machines that used gamma rays from cobalt-60 to treat cancer cells (1.2 MV).

### The First Radiation Oncologist in Jordan

The MOH had no qualified radiation oncologists until 1974 when the International Atomic Energy Agency hired a Polish radiation oncologist to work at Al Basheer Hospital. He contributed to the creation of a separate radiation oncology department in 1978 and ran the cancer RT services at the hospital until 1984.

The need for qualified graduates in radiation oncology led to the creation of the first residency program in Jordan, which began in 1987 when the first resident joined the newly created radiation oncology residency program at Al Basheer Hospital. The program is the oldest in Jordan and graduates 2 to 3 residents every year. Residents attend weekly and monthly lectures and are evaluated annually and at the end of their residency by taking the medical council board examination.

By 1987, 6 new qualified radiation oncologists who spent their residency training abroad in Russia, the United Kingdom, and Spain returned to Jordan. Three of them joined the MOH Radiation Oncology Department and 2 joined the RMS. The remaining radiation oncologist worked in the private sector mainly as a clinical oncology practitioner. In 1987, the RT services at the hospital were expanded once more by the addition of the first linear accelerator (LINAC) machine (Varian). Two new EBRT cobalt-60 machines were also installed at Al Basheer Hospital.

### KHCC

The inauguration of the KHCC in 1997 was a major boost to the already growing radiation oncology practice in Jordan. The KHCC received Disease-Specific (Oncology) Certification from Joint Commission International.^6^ The newly created Radiation Oncology Department had 4 new state-of-the-art LINACs and a computed tomography (CT) simulator for treatment planning. The center was also the first to be able to offer brachytherapy in Jordan by acquiring a machine for administering high-dose iridium-192 (Ir-192) brachytherapy. At that time, the department had 3 radiation oncologists and other supporting staff (physicists, technologists, and medical engineers). But by 2004, the need arose for a complete residency program that could match the standards of its peers in the developed world. This was accomplished by creating the KHCC Radiation Oncology Residency Program.

By implementing its own residency program (which adheres to the tenets of competence-based medical education), the KHCC was able to increase its faculty from 3 senior radiation oncologists in 2004 to 17 in 2018. The 4-year residency program at KHCC is complemented by a 3-month externship at a partner cancer center in the United States or the United Kingdom. Residents are required to sit for the American College of Radiologists examinations every year and must take the Medical Council board examination at years 2 and 4 of their residency. Forty-one Jordanian and non-Jordanian residents had successfully completed their residency program by 2019. Currently, there are 17 radiation oncologists and 13 residents at KHCC.^7^

### New Centers Enter Service

In 2006, the Al Afia Radiotherapy and Nuclear Medicine Center (a private radiation oncology facility) was established in Amman. The Center started its treatment services with one LINAC and a CT simulator. Its staff consisted of 3 radiation oncologists, 2 physicists, and 2 technologists. In 2008, another LINAC was added, and the number of technologists increased to 6. The Jordanian RMS has expanded its efforts from providing medical services to the Jordanian military, so it now includes civilians and provides them with high-quality medical service. In 2012, a new radiation oncology department was established at Queen Alia Hospital, RMS. The newly created department has 2 LINACs and a CT simulator. In 2014, a high dose rate (HDR) system was installed to administer brachytherapy.

At its creation, the department had 3 radiation oncologists, 6 technologists, 2 physicists, and 1 medical engineer. The residency program at Queen Alia Hospital started in 2014. The program graduates 2 to 3 residents every year. Residents are required to attend weekly and monthly lectures by the department faculty and sit for annual examinations. By 2019, the department staff had increased to 9 radiation oncologists, 14 technologists, 9 physicists, and 1 medical engineer, making it one of the largest in Jordan.

## CURRENT STATE OF RADIATION ONCOLOGY IN JORDAN AND FUTURE CHALLENGES

Over the past 15 years, the incidence of cancer in Jordan has risen steadily. A 2018 epidemiologic study reported a 60% overall increase in the incidence of cancer cases for the period of 2000 to 2013.^8^ Currently, there are 4 centers that provide RT in Jordan: (1) Al Basheer Hospital, (2) Queen Alia Hospital, RMS, (3) KHCC, and (4) Al Afia Radiotherapy and Nuclear Medicine Center. Tables 1 and 2 list the staff and available equipment for each of these centers.

**TABLE 1:**
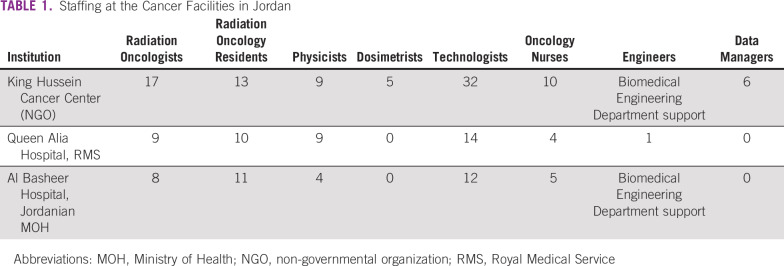
Staffing at the Cancer Facilities in Jordan

**TABLE 2:**
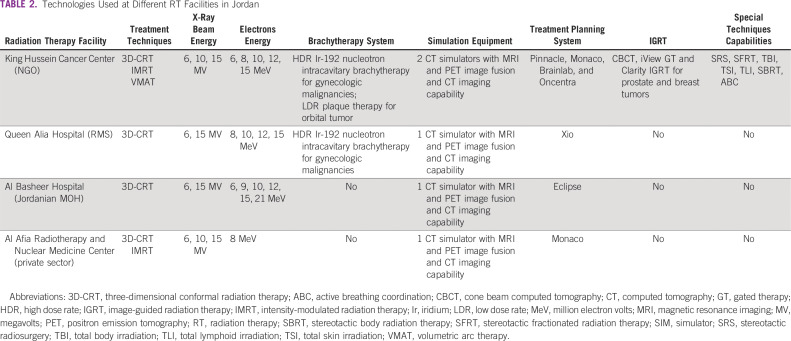
Technologies Used at Different RT Facilities in Jordan

**FIG 2:**
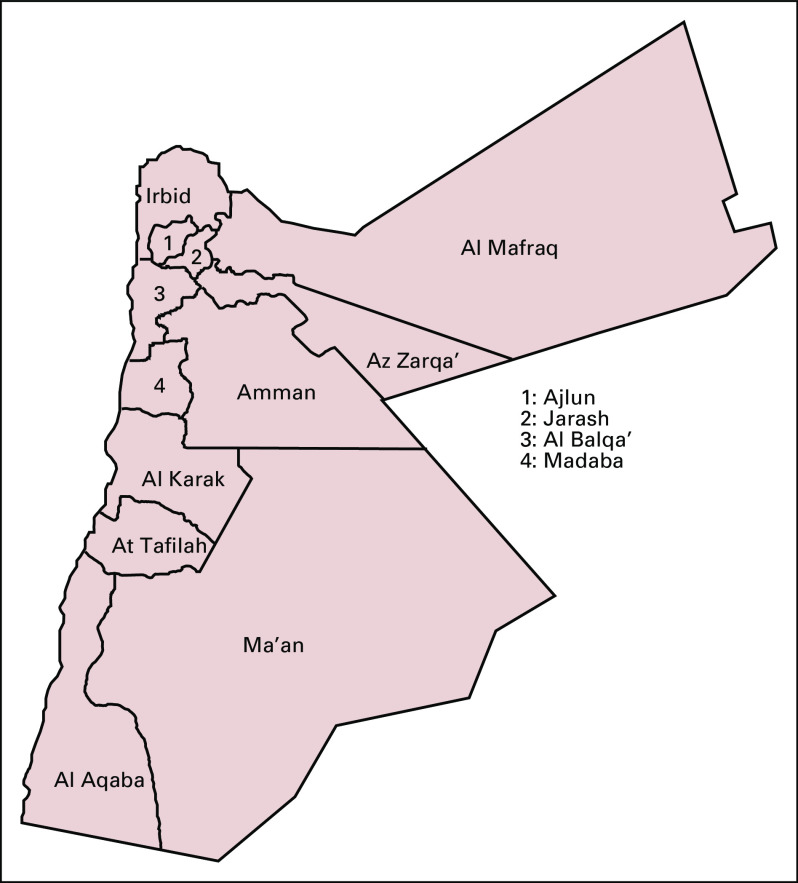
Jordan map showing governorates.

Jordan’s total surface area is 89,320 km^2^, which means that each center should theoretically cover the population in a land area of 22,230 km^2^. Unfortunately, all 4 centers are located in Amman in central Jordan (home to about 4.2 million people). Because the treatment centers are all concentrated in one central area, about 6 million people in the north and south governorates of the country have to travel long distances to receive treatment (Fig 2). The distribution of the population in Jordan across different governorates is presented in Table 3.

**TABLE 3:**
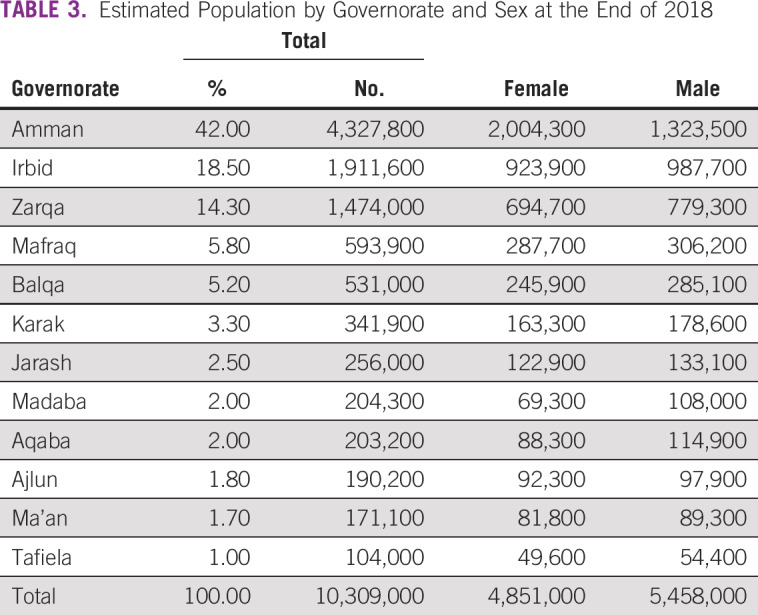
Estimated Population by Governorate and Sex at the End of 2018

RT machines are complicated and highly technical machines that are expensive and difficult to operate. EBRT or teletherapy is the most frequently used form of RT. The traditional EBRT cobalt-60 machine was the main teletherapy equipment available in Jordan until recently when LINACs took over. A LINAC enables us to use various means to modify the shape and intensity of the beam it produces so we can produce different types of RT (conventional, conformal, intensity-modulated, tomographic, and stereotactic) according to patient needs.^9^ There are currently 12 LINACs in Jordan (6 at KHCC, 2 at Al Basheer Hospital, 2 at Al Afia Radiotherapy and Nuclear Medicine Center, and 2 at Queen Alia Hospital) with a population coverage of 830,000 MV.

The low number of machines available combined with the high patient load means that machines at all 4 centers frequently break down. The KHCC Radiation Oncology Department treats 2,300 patients every year, and the Al Basheer Hospital (MOH) treats 1,500 patients per year. This puts the machines at Al Basheer Hospital under serious strain because there is only 1 machine available for treating 750 patients compared with KHCC’s 1 machine for treating 384 patients, which is still a high number. The low number of available machines compels us to use them frequently, and even with attentive maintenance, breakdowns happen, which means that several treatment plans have to be delayed.

Brachytherapy is a form of RT for small and deep tumors.^10^ It involves inserting a device into a body cavity (intracavitary brachytherapy) or into a lumen (intraluminal brachytherapy) or directly into body tissues (interstitial brachytherapy). Currently, KHCC is the only center that provides brachytherapy. KHCC provides intracavitary HDR brachytherapy for gynecologic and nasopharyngeal malignancies, and low-dose rate (LDR) brachytherapy for tumors of the eye. Recently, a new brachytherapy system was installed at Queen Alia Hospital (RMS).

Other sophisticated modalities that require a high level of technical expertise include three-dimensional conformal RT (3D-CRT), intensity-modulated RT (IMRT), image-guided RT (IGRT), and stereotactic radiosurgery (gamma knife).^11^ Figure 1 shows the timeline for the evolution of different radiation techniques. Advanced RT modalities are lacking in Jordan. The main treatment modality at the 4 centers is the 3D conventional RT. IGRT is available at KHCC and Al Afia Radiotherapy and Nuclear Medicine Center, but brachytherapy treatment is available only at KHCC and Queen Alia Hospital (RMS).

KHCC leads the way in improving its inventory of these different systems. Advanced technologies have been acquired and implemented there, and the experience of practitioners has been harnessed so that other centers can benefit from it. KHCC currently has the only IGRT unit in Jordan. In addition, the center has other sophisticated modalities such as stereotactic radiosurgery, stereotactic body radiosurgery, total body irradiation, total skin irradiation, and an active breathing control system for breast RT.^12,13^ Considering the costs of all of the various treatment modalities used for cancer care, RT represents around 5%.^14^ In Jordan and other Arab countries, RT composes 5% to 10% of collective cancer treatment costs. A typical course of 3D-CRT (eg, for breast cancer treatment) costs $2000 to $6000, whereas IMRT or volumetric modulated arc therapy (VMAT) cost $3,250 to $11,500, which parallels the costs in neighboring countries (Table 4).^15^ Compared with costs for these treatments in Western countries, ours are almost half price.^16^

**TABLE 4:**
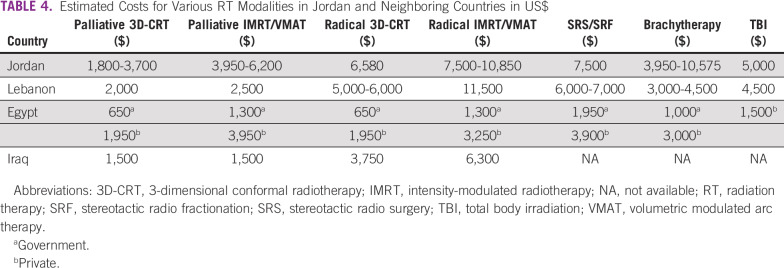
Estimated Costs for Various RT Modalities in Jordan and Neighboring Countries in US$

### Future Plans

There has been a growing interest in addressing the deficiency in equipment and trained personnel by the Jordanian government, RMS, and the private sector. By increasing financial allocations, charity fundraising, and private sector contributions, radiation oncology has grown as a specialty in Jordan in the past 20 years.

The increasing need for RT for different types of malignancies necessitates a greater effort to create new centers with state-of-the-art equipment and highly trained radiation oncologists and supporting staff. King Abdullah University Hospital in Irbid (north Jordan) has taken the initiative. A new radiation oncology department will be created there to serve the local population and to increase the pool of machines that can provide RT in Jordan overall. The new department will have two LINAC, one CT simulator, and one HDR brachytherapy unit.

Similarly, the existing centers plan to expand their inventory of equipment and staffing capacity. KHCC intends to install 2 new LINACs and 1 magnetic resonance imaging–based simulator. Al Basheer Hospital is expanding into a new building that has 2 new LINACs capable of IMRT and VMAT techniques. Queen Alia Hospital will also install a new LINAC for both techniques.

In conclusion, because Jordan is a small developing country with a low gross domestic product, it faces numerous challenges in maintaining viable RT services. Efforts since the late 1950s have resulted in an ever-growing service with a total of 4 centers. The difficulties of distributing centers evenly throughout the population and the low number of machines are currently the greatest obstacles. However, we aspire to maintain the growing interest in providing affordable and high-quality RT services that serve the Jordanian population and neighboring Arab countries.
